# Transcriptional Profiling of *Wnt3a* Mutants Identifies Sp Transcription Factors as Essential Effectors of the Wnt/β-catenin Pathway in Neuromesodermal Stem Cells

**DOI:** 10.1371/journal.pone.0087018

**Published:** 2014-01-24

**Authors:** William C. Dunty, Mark W. L. Kennedy, Ravindra B. Chalamalasetty, Kenneth Campbell, Terry P. Yamaguchi

**Affiliations:** 1 Cancer and Developmental Biology Laboratory, Center for Cancer Research, NCI-Frederick, NIH, Frederick, Maryland, United States of America; 2 Division of Developmental Biology, Cincinnati Children’s Hospital Medical Center, Cincinnati, Ohio, United States of America; University of Minnesota, United States of America

## Abstract

Neuromesodermal (NM) stem cells reside in the primitive streak (PS) of gastrulating vertebrate embryos and generate precursors of the spinal cord and musculoskeletal system. Although Wnt3a/β-catenin signaling is crucial for NM stem cell maintenance and differentiation, few key transcriptional effectors have been identified. Through a concerted transcriptional profiling and genetic approach we have determined that two Zn^2+^-finger transcription factors, *Sp5* and *Sp8,* are regulated by Wnt3a in the PS, and are essential for neural and musculoskeletal patterning. These results identify *Sp5* and *Sp8* as pivotal downstream effectors of Wnt3a, and suggest that they are essential for the self-renewal and differentiation of NM stem cells.

## Introduction

Germ layer specification is a fundamental aspect of embryogenesis that occurs in the PS region during gastrulation. Under the influence of signals emanating from the PS, pluripotent epiblast stem cells differentiate into the multipotent progenitors that will form the germ layers, ie. the ectoderm, mesoderm and endoderm. As development proceeds, the differentiation potential of descendants of these early embryonic stem cells becomes progressively restricted [1]. For example, the descendants of NM stem cells, which originate in the epiblast adjacent to the PS, become restricted to the multipotent neuroectoderm and paraxial mesoderm cell lineages upon differentiation [2]–[6]. The response of NM stem cells to signals secreted by the PS, such as Wnt3a, determines whether they self-renew or differentiate into these lineages, however the mechanisms that govern NM cell fate decisions are not well understood.

Wnt3a is a member of the ‘Wnt’ family of secreted glycoproteins that interact with Frizzled/Lrp receptor complexes to prevent the proteolytic degradation of the transcriptional co-activator β-catenin. In the presence of Wnt3a, stabilized β-catenin enters the nucleus where it interacts with members of the Tcf/Lef family of DNA binding transcription factors. Together, β-catenin and Tcf/Lef form the core of a multipartite transcription complex that controls target gene expression [7]. Embryos lacking *Wnt3a* die at mid-gestation with severe posterior axial truncations that reflect a loss of neuro-musculoskeletal progenitors [4], [8]–[12]. Cells expressing the stem cell marker and T-box transcription factor, *T*, including NM stem cells, are transiently established in *Wnt3a^−/−^* mutants but are not maintained [10]. Interestingly, *Wnt3a^−/−^* mutants form ectopic neural tubes that are speculated to arise from the transfating of paraxial mesoderm progenitors in the absence of a Wnt signal [10], [12]. Similar defects are also observed in *Tcf1^−/−^;Lef1^−/−^* embryos [13]. Together, these findings strongly suggest that Wnt3a/β-catenin signaling regulates the maintenance and differentiation of NM stem cells. A thorough understanding of the target genes and the gene regulatory network controlled by Wnt3a will provide crucial insights into how the fundamental neural and paraxial mesoderm lineages are established.

Here we report the comprehensive analysis of the *Wnt3a*-dependent transcriptome in PS and node stem cells. *In silico* analysis demonstrates that *Wnt3a* regulates the expression of many genes associated with the primitive streak, neuroectoderm and paraxial mesoderm. Our analysis identified 2 candidate target genes, *Sp5* and *Sp8*, that when genetically disrupted recapitulate the *Wnt3a* null phenotype. Thus, *Sp5* and *Sp8* are redundant factors that function as key effectors of the Wnt3a/β-catenin pathway in NM stem cells.

## Materials and Methods

### Mice

*Ctnnb1^tm2Kem^*, *Ctnnb1^lox(ex3)^*, *Wnt3a, T-cre*, *Sp5^lacZ/lacZ^* and *Sp8* ^Δ*/+*^ mice were described previously [10], [14]–[18]. *T-cre* mice were used to conditionally delete *Ctnnb1^tm2Kem^* or *Ctnnb1^lox(ex3)^* in NM stem cells and their progeny. *Sp5;Sp8* dko embryos were obtained by intercrossing *Sp5^lacZ/lacZ^;Sp8* ^Δ*/+*^mice. This study was carried out in strict accordance with the recommendations in the Guide for the Care and Use of Laboratory Animals of the National Institutes of Health. The protocol was approved by the Frederick National Lab Animal Care and Use Committee (Animal Study Proposal #12-408). All efforts were made to minimize suffering. Rodents were euthanized by CO2 inhalation in accordance with the most recent AVMA Guidelines on Euthanasia.

### Gene Expression Profiling

Total RNA was isolated from dissected node and PS regions of E7.75-E8 wildtype and *Wnt3a* mutant embryos as previously described [15]. cDNA and cRNA synthesis, hybridization to GeneChip® Mouse Genome 430 2.0 Arrays, washing, staining, and array scanning were carried out according to manufacturer’s protocols (Affymetrix, Santa Clara, CA) as previously described [15], [19]. Briefly, 30–50 ng of total RNA from individual embryos was used as template for first cycle cDNA synthesis. For second cycle cDNA synthesis, equivalent amounts of cRNA from three stage-matched embryos per genotype were pooled (600 ng total) to generate labeled-target per array. Array hybridizations were performed in triplicate per genotype.

**Figure 1:**
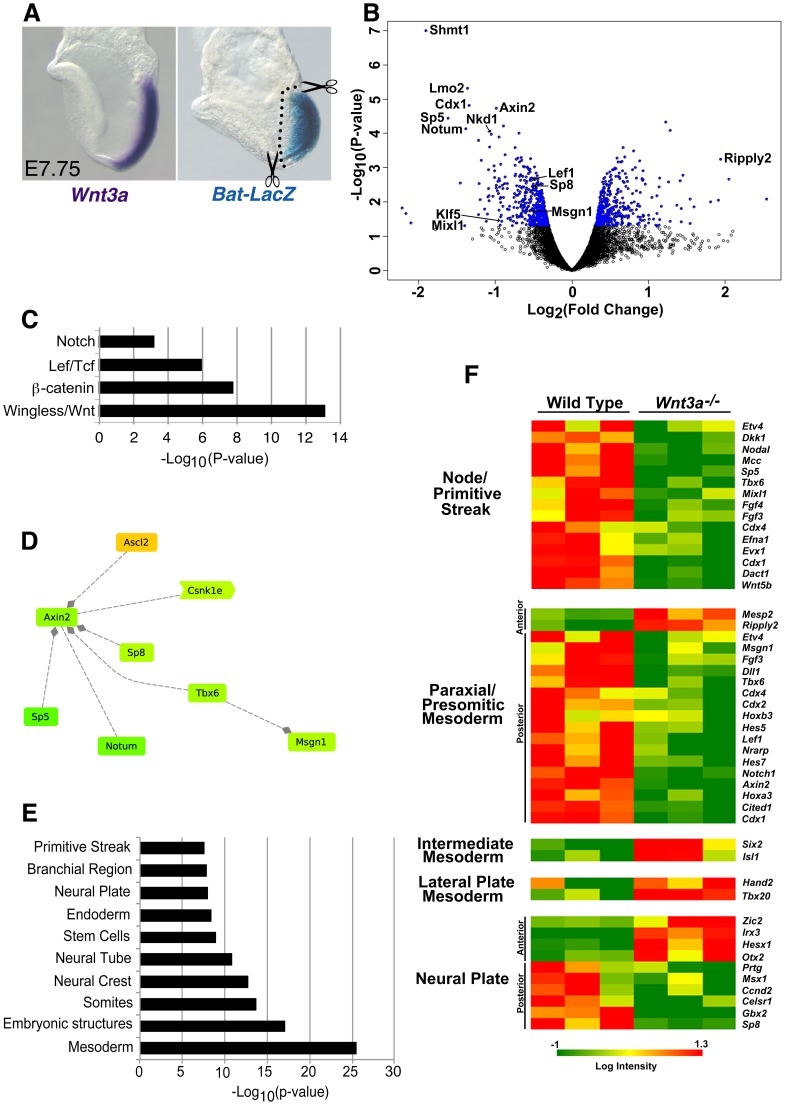
Transcriptional profiling of wild type and *Wnt3a^−/−^* embryos. A) WISH of a wt embryo at E7.75 illustrating *Wnt3a* expression in the PS and node (left panel). (right panel) The corresponding domain of Wnt/β-catenin activity as assessed by X-gal staining of the transgenic Wnt/β-catenin reporter, BAT-lacZ [15]. Dotted lines demarcate dissection boundaries. B) Volcano plot depicting all statistically significant DEGs (p<0.05, indicated by blue color). C–E) Gene ontology (GO) analysis of DEGs identified a highly significant association with the Wnt/β-catenin pathway (C, D) and with expression in embryonic mesoderm, somites and neural tissue (E). D) A selection of the shortest path network representation of Wnt pathway genes in (C) analyzed in Genomatix GePS. For complete network see Figure S2. F) Analysis of tissue-specific DEGs shows that posteriorly restricted genes expressed in the node/PS, PSM, and NP are down-regulated while anterior PSM, anterior NP, IM, and LPM genes were up-regulated. Green: down-regulated genes; Red: up-regulated genes.

Statistical analysis was performed on probe-intensity level data (CEL files) using BRB ArrayTools version 4.3.0 or GeneSpringGX software, with similar results. Data presented herein was generated using BRB ArrayTools. Genes were considered statistically significant if their parametric p-value ≤0.05. For hierarchical clustering of genes in Fig. 1F, centered correlation and average linkage method was used for the maximally expressed probe set measured by average intensity across all arrays. Signal transduction and tissue pathway analysis was performed using Genomatix suite (GePS) software. The microarray data in this study was previously deposited in the GEO database under accession code GSE29995 [19].

### *In situ* Hybridization, Immunofluorescence and Tissue Sectioning

*Brachyury*, *Wnt3a* and *Uncx4*.*1* probe synthesis and whole-mount in situ hybridizations (WISH) were performed as previously described [15]. The mouse *Sp5* cDNA (cat# EMM1002-5885071) was obtained from Open Biosystems. The mouse *Sp8* DNA template was obtained by TA-TOPO cloning (Invitrogen) the *Sp8* coding region from ES cell cDNA. Paraffin and cryosectioning was performed at the Pathology and Histology Laboratory (PHL, NCI-Frederick). Cryosections were washed in 1×PBT (1×PBS +0.1% tween-20), incubated in 1×PBS +0.2% Triton-X for 20 minutes, washed in PBT. Sections were incubated in primary antibodies in 0.02% block (Roche Blocking reagent in PBT, filtered) overnight at 4°C. Sections were then washed 3 times in PBT. Secondary antibodies in 0.02% block were applied at room temperature for 2 hours. Sections were washed 3 times, 1 nM DAPI (4′,6-diamidino-2-phenylindole) was included in the second wash for 20 minutes. Embryos/sections were photographed on a Leica MZFLIII stereoscope equipped with a Zeiss Axiovision HRc digital camera, and Zeiss Axiovision imaging software (v4.5). Unless indicated otherwise, at least 3 mutant embryos were examined with each probe/antibody, and all yielded similar results.

### ES Cell Culture and mRNA Expression Analysis

T-GFP ES cells were maintained on gelatin-coated plates and differentiated as embyoid bodies (EBs) in serum-free, feeder-free conditions, as previously described [20]. Day 2 EBs were dissociated with Trypsin-EDTA and 4×10^5^ cells were cultured in ultra-low attachment 6-well dishes with 100 ng/ml recombinant Wnt3a (R&D Systems) or 1 µM CHIR99021 (ToCris) for 2 days. Total RNA was extracted using the RNeasy miniprep kit (Qiagen) and first stand cDNA synthesized using iScript cDNA synthesis kit (Quanta Biosciences). Relative gene expression was analyzed with iTaq universal SYBR Green (BioRad) using the CFX96 Real-Time PCR Detection system (BioRad). Relative fold changes were calculated by normalizing to *Gapdh* levels using the 2^−ΔΔCt^ method. qPCR primers are listed in Table S4.

## Results

### Transcriptional Profiling of PS and Node Stem Cells

*Wnt3a* is highly expressed in the node and primitive streak during gastrulation, where it signals via β-catenin and Tcf1/Lef1 (Fig. 1A; [15]) to regulate genes essential for neuromesodermal development. To identify the effectors of this pathway, we used microarrays to generate transcriptional profiles of wild type and *Wnt3a^−/−^* primitive streak and node tissue. To enrich for early molecular regulators, tissues were excised between E7.75–8.0 when *Wnt3a^−/−^* mutants are morphologically indistinguishable from wild type controls. Comparisons of wild type and mutant transcriptional profiles identified 729 differentially expressed genes (DEGs, p<0.05) of which 379 were down-regulated and 350 were up-regulated (Fig. 1B, Table S1 and Figure S1). Included amongst the down-regulated genes are several well-known targets that encode components of the Wnt/β-catenin signaling pathway itself, ex. *Axin2, Nkd1,* and *Lef1* (Fig. 1B, Table S1), thereby providing an initial validation of the screen [21]–[23]. An assessment of DEGs for their association with signaling pathways revealed that the Wnt/β-catenin pathway was most highly represented followed closely by the Notch, Hedgehog, and Fgf pathways (Fig. 1C, Table S2). The DEG data was mined further for associations with literature-based gene regulatory networks using Genomatix Pathway Systems software. This approach identified an interacting network of Wnt/β-catenin pathway genes that function, in part, to control the expression of *Axin2* (Fig. 1D, Fig. S2). Included in this network of Wnt target genes are the transcription factors *Msgn1* and *Tbx6* that play critical roles in paraxial mesoderm and neural development [19], [24], [25]. Interestingly, two members of the large and well-characterized Sp1 family of DNA-binding Zn^2+^-finger transcription factors, *Sp5* and *Sp8*, were also identified in this network.

We then asked if these DEGs are expressed in the appropriate embryonic tissues to be targets of Wnt3a. Detailed analysis of the DEGs using their associated gene ontology (GO) terms revealed that, as expected, their expression is most commonly associated with stem cells, the PS, neural plate, and mesoderm (Fig. 1E, Table S3). Analysis of genes clustered by their tissue-specific expression patterns revealed that primitive streak and posterior PSM genes were down regulated in *Wnt3a^−/−^*embryos, while anterior PSM genes were increased (Fig. 1F). These results are consistent with Wnt3a maintaining immature posterior PSM progenitors, while suppressing segmentation genes in the anterior PSM [19], [26]. Interestingly, intermediate and lateral plate mesoderm genes showed increased expression levels in the mutants suggesting *Wnt3a* has a mesodermal patterning role in the PS. It is also notable that anterior neural plate markers increased, but posterior neural markers decreased in the Wnt3a mutants, likely reflecting the established neural posteriorizing role for Wnt signaling [27], [28].

### Validation of Candidate *Wnt3a* Target Genes

**Figure 2:**
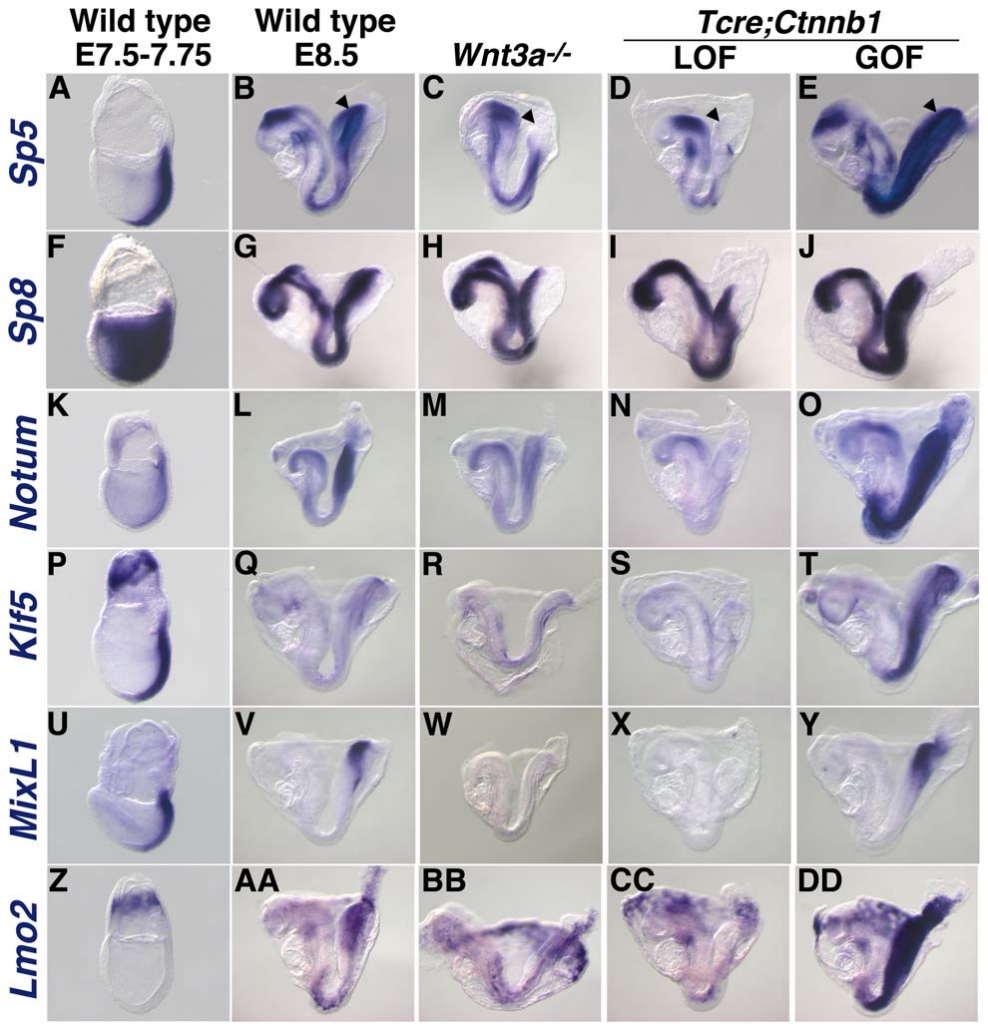
Validation of candidate *Wnt3a* target gene expression in *Wnt3a* and *Ctnnb1* mutants by WISH. A comparison of *Sp5* (A–E), *Sp8* (F–J), *Notum* (K–O), *Klf5* (P–T), *MixL1* (U–Y), and *Lmo2* (Z-DD) expression in wild type (A, B, F, G, K, L, P, Q, U, V, Z and AA), *Wnt3a^−/−^* (C, H, M, R, W and BB), *Tcre;Ctnnb1^flLOF^* (D, I, N, S, X and CC) and *Tcre;Ctnnb1^flGOF^* (E, J, O, T, Y and DD) embryos at E7.5 and 8.5 (LOF: loss-of-function; GOF: gain-of-function). Arrowhead in B–E points to the PS.

We focused on the validation of putative Wnt3a target genes that encode transcription factors, as they frequently function as master regulators of stem cell fate. Microarray analysis showed that the *Sp5, Sp8, Klf5, Mixl1,* and *Lmo2* transcription factors were all down-regulated in *Wnt3a* mutants, and were thus selected for further expression analysis *in vivo*, along with the secreted Wnt feedback inhibitor *Notum*. We reasoned that candidate Wnt3a target genes should be spatially and temporally co-expressed with *Wnt3a* and be responsive to changes in Wnt3a/β-catenin signaling. WISH analysis demonstrated that *Sp5, Sp8, Notum, Klf5,* and *Mixl1* were co-expressed with *Wnt3a* (Fig. 1a, left) in the wild type node/primitive steak region at E7.5–8.5 (Fig. 2A, B, F, G, K, L, P, Q, U, V). *Lmo2* was undetectable in the PS at E7.5, but was easily detected at E8.5 (Fig. 2Z, AA). The lack of *Lmo2* expression at E7.5, when *Wnt3a* is easily detected in the PS, suggests that *Lmo2* requires additional signals for activation. Reduced Wnt3a/β-catenin activity led to significantly diminished expression of all 6 genes in the PS and posterior mesoderm domains of *Wnt3a^−/−^* embryos *(* Fig. 2C, H, M, R, W, BB), and in embryos conditionally lacking *Ctnnb1* (β-catenin) activity in mesoderm progenitors (Fig. 2D, I, N, S, X, CC). Correspondingly, these genes were all highly up-regulated in the PS and PSM of embryos in which β-catenin was constitutively activated in mesoderm progenitors (Fig. 2E, J, O, T, Y, DD). Thus, all 6 genes lie downstream of Wnt3a and β-catenin *in vivo*, thereby validating the microarray results and furthering their candidacy as effectors of the Wnt3a/β-catenin signaling pathway.

### *Sp5* and *Sp8* are *Wnt3a*-responsive and Expressed in the PS

**Figure 3:**
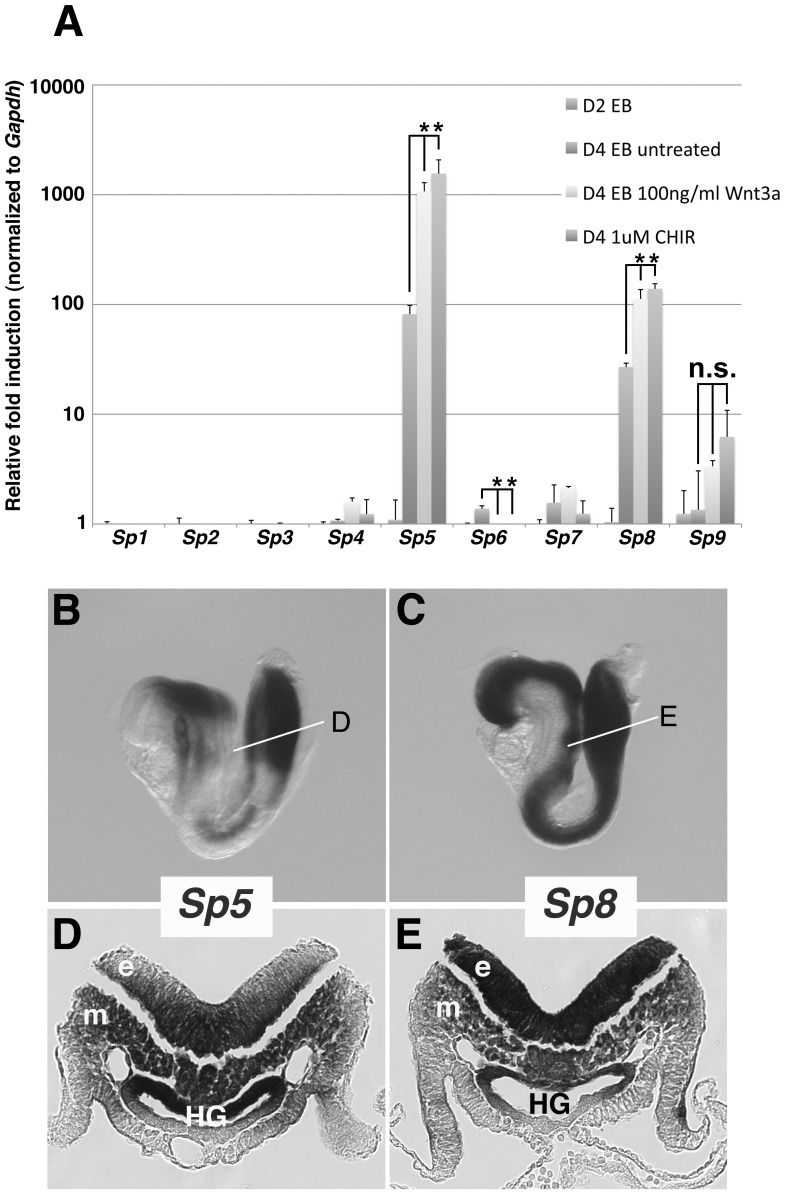
Analysis of *Sp1*-like family members for co-expression and Wnt3a responsiveness. A) qPCR analysis of the *Sp1*-like family mRNA levels in ESCs stimulated with recombinant Wnt3a protein or the GSK3β inhibitor CHIR99021 on day 2 (D2) and assayed on day 4 (D4). mRNA levels were normalized to *Gapdh* expression. Values represent the mean, calculated from 3 independent qPCR reactions. Error bars, one standard deviation; *, statistical significance (p<0.05, 2-tailed students t-test); n.s., not significant. WISH of *Sp5* (B) and *Sp8* (C) at E8.5 illustrates the overlapping expression in the midbrain and the posterior of the embryo. (D, E) Sections generated at the positions indicated by lines in (B) and (C) show *Sp5* and *Sp8* co-expression across all three germ layers. Ectoderm (e), Mesoderm (m), and Hindgut (HG).

*Sp5* and *Sp8* were identified by microarray as two of the more prominently down regulated genes in *Wnt3a* mutants. The lack of a phenotype in *Sp5* null mice, taken together with the observation that *Sp1, Sp5,* and *Sp8* all bind to the same DNA sequence *in vitro* [17], [29], [30], suggests that Sp factors could function redundantly during development. To determine whether additional members of the Sp family are expressed in the PS and are Wnt3a-responsive, we turned to an *in vitro* mouse embryonic stem cell (ESC) model of PS formation [20]. ESCs were cultured in defined media to allow for the stimulation of the Wnt/βcatenin signaling pathway with purified, recombinant Wnt3a protein in feeder-free and serum-free conditions. ESCs were primed for germ layer differentiation by withdrawing LIF and forming embryoid bodies (EBs) for 2 days. EBs were then treated with Wnt3a or the GSK-3β inhibitor CHIR99021 for an additional 2 days to promote PS and mesoderm gene expression [20], [31], [32]. Quantitative PCR of EBs revealed that of the 9 *Sp* genes (*Sp1-9*), only *Sp5* and *Sp8* were expressed during the in vitro differentiation process (Fig. 3A, D4 EB untreated). These expression levels were dramatically augmented by treatment with Wnt3a protein or CHIR99021 (Fig. 3A) demonstrating that *Sp5* and *Sp8* were induced during PS formation and were highly Wnt3a-responsive.

To examine whether *Sp5* and *Sp8* are expressed in the PS *in vivo*, a detailed comparative analysis was performed on serial sections of E8.5 embryos processed for WISH (Fig. 3B, C). Section analysis shows definitively that *Sp5* and *Sp8* are both expressed in the PS and across all three germ layers. In addition to the expression in the PS ectoderm and mesoderm, *Sp5* is highly expressed in the dorsal hindgut (Fig. 3D). *Sp8* is weakly expressed in the dorsal hindgut but is highly expressed in the PS epiblast/caudal neural plate (Fig. 3E). Together, these results demonstrate that *Sp5* and *Sp8* respond to Wnt3a/β-catenin activity, and are co-expressed in the PS, including the NM stem cells in the caudal lateral epiblast.

### *Sp5* and *Sp8* Function in the *Wnt3a* Genetic Pathway

**Figure 4:**
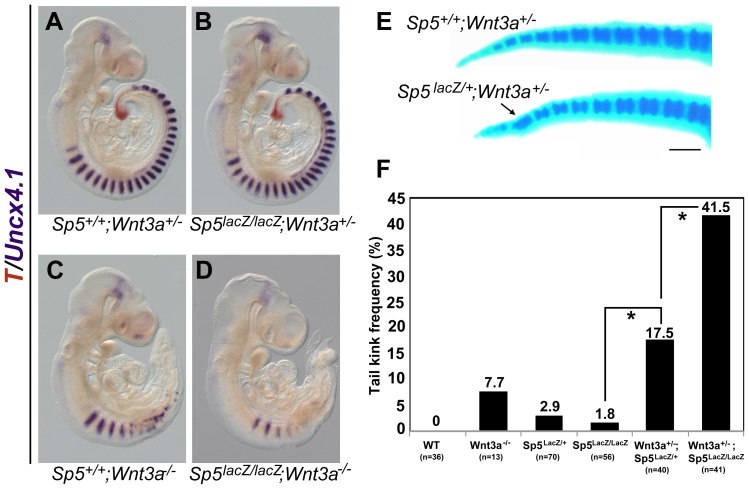
Genetic interactions between *Sp5* and *Wnt3a* during tail development. (A–D) Two-color WISH illustrating *Uncx4.1* (purple) and *T* (orange) in *Sp5* ^+/+^;*Wnt3a* ^+/−^ (A), *Sp5* ^lacz/lacz^;*Wnt3a* ^+/−^ (B), *Sp5* ^+/+^;*Wnt3a* ^−/−^ (C), and *Sp5* ^lacz/lacz^;*Wnt3a* ^−/−^ double mutants (D) at E9.5. Double mutants (*n* = 8) display fewer and less-defined *Uncx4.1^+^* anterior somites as compared to stage-matched *Wnt3a* null embryos. (E) At E18.5, *Sp5* ^lacz/+^;*Wnt3a* ^+/−^ animals (bottom) display abnormal, fused tail vertebrae (arrow) compared to *Sp5* ^lacz/+^;*Wnt3a* ^+/−^ mice (top). (F) Frequency of tail kinks (expressed as %) observed in *Wnt3a* ^+/−^ heterozygotes at 6 weeks of age increased dramatically as the *Sp5* gene dosage was reduced. The number of animals examined (n) is indicated below each genotype. *, statistical significance (p<0.0001, Chi-squared t-test). Scale bar = 500 um (E).

Our bioinformatics analysis and ESC experiments suggest that *Sp5* and *Sp8* could participate in Wnt signaling. To address this hypothesis we first asked if *Sp5* genetically interacts with *Wnt3a*. Although *Sp5^LacZ/LacZ^* null mice are viable and fertile, both homozygotes and heterozygotes develop “kinked” tails at a frequency of ∼2–3% (Fig. 4E–F and Fig. S3). This phenotype is observed at a similar frequency in *Wnt3a* heterozygotes (7.7%, Fig. 4F) and is indicative of defective somite formation leading to fused vertebrae [33]. The frequency of this tail defect rises to 17.5% in *Sp5^LacZ/+^;Wnt3a^+/−^* mice and 41.5% in *Sp5^LacZ/LacZ^;Wnt3a^+/−^* animals demonstrating an *Sp5* gene dosage-dependent genetic interaction with *Wnt3a* during tail development (Fig. 4E–F). Similar genetic interactions have been reported for *Sp5* and the Wnt3a target gene *T* [11], [17]. Furthermore, *Sp5^LacZ/LacZ^;Wnt3a^−/−^* double mutants display a loss of caudal somites and tailbud that is slightly more severe than *Wnt3a^−/−^* mutants alone (Fig. 4A–D). These results support a role for *Sp5* in segmentation and the maintenance of tail progenitors and suggest that *Sp5* functions in a genetic network with *Wnt3a* and *T*.

**Figure 5:**
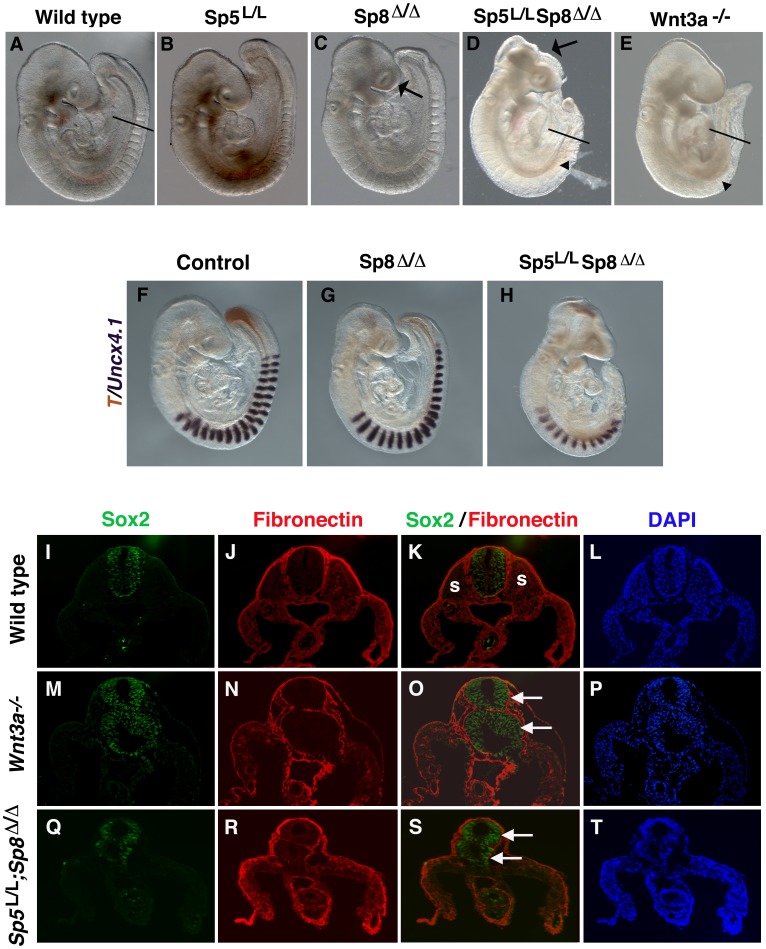
*Sp5;Sp8* double mutants phenocopy single *Wnt3a^−/−^* mutants. Brightfield images depicting wild type (A), *Sp5^LacZ/LacZ^* (B) *Sp8* ^Δ*/*Δ^, (C), *Sp5^LacZ/LacZ^;Sp8* ^Δ*/*Δ^ (D), and *Wnt3a^−/−^* (E) mutant phenotypes at E9.5. Arrows in (C) and (D) indicates the open anterior neuropore. Arrowheads in (D) and (E) indicate position of last formed somite. (F–H) Two-color WISH analysis of somite polarity (*Uncx4.1)* and tailbud progenitor (*T)* markers in wt and mutant embryos at E9.5. F) Normal *T* expression in the PS and posterior PSM in control embryos. *T* was severely down regulated in *Sp8* ^Δ*/*Δ^ embryos (G), and completely absent from *Sp5^LacZ/LacZ^;Sp8* ^Δ*/*Δ^ mutants (H). (I–T) Immunofluorescence analysis of Sox2 and Fibronectin protein expression in wild type (I–L), *Wnt3a^−/−^* (M–P), and *Sp5^LacZ/LacZ^;Sp8* ^Δ*/*Δ^ (Q–T) mutants. Note the absence of somites and the duplicated neural tubes (arrows) in the *Wnt3a^−/−^* and *Sp5^LacZ/LacZ^;Sp8* ^Δ*/*Δ^ mutants. Nuclei were labeled with DAPI. Approximate positions of sections are indicated by lines in (A), (D) and (E). Somites (s).

*Sp8* null embryos are tailless and display spina bifida, reflecting a failure to form caudal somites and maintain a tailbud, and an eversion of the posterior neural folds, respectively [14], [34]. Interestingly, the *Sp8* ^Δ*/*Δ^ axis truncation phenotype bears a striking resemblance to the tail and spina bifida phenotypes observed in the *Wnt3a* hypomorph, *vestigial tail (vt)* [33]. The similarities between the hypomorphic *Wnt3a* and *Sp8* mutant phenotypes, together with the demonstration that *Wnt3a* and *Sp5* function in the same genetic pathway and that *Sp5* and *Sp8* are co-expressed in the PS, strongly suggests that *Sp5* and *Sp8* could function redundantly in the *Wnt3a* pathway to regulate NM stem cells. To test this hypothesis, we generated *Sp5^LacZ/LacZ^;Sp8* ^Δ*/*Δ^ double knockout (hereafter referred to as *Sp5;Sp8* dko) embryos. At E9.5, *Sp5^LacZ/LacZ^* and *Sp8* ^Δ*/*Δ^ single mutants appear similar to wild type controls (Fig. 5A–C). Although *Sp5^LacZ/LacZ^;Sp8* ^Δ*/+*^ mice are viable and fertile, 33.3% of adults develop “kinked” and/or shortened tails (Fig. S3). In contrast, tail defects were observed in only ∼2.2% of *Sp8* ^Δ*/+*^ mice, suggesting a requirement for both *Sp5* and *Sp8* in tail segmentation and extension. Analysis of *Sp5;Sp8* dko revealed embryonic lethality by ∼E13.5–14.5. Remarkably, *Sp5;Sp8* dko embryos displayed a severe posterior mesoderm deficit at E9.5 (Fig. 5D) that closely resembled the phenotype observed in *Wnt3a^−/−^* mutants (Fig. 5E). Although *Sp5;Sp8* dko formed ∼12 somites, compared to the 7–10 somites that typically develop in *Wnt3a^−/−^* mutants, the *T^+^* NM stem cell population is completely depleted in *Sp5;Sp8* dko by E9.5, just like in *Wnt3a^−/−^* mutants (Fig. 5F–H compared to Fig. 4C). It is notable that the open anterior neuropore that was present in all *Sp8* nulls (n = 28, Fig. 5C arrowhead) was exacerbated in 17/31 double mutants (arrow, Fig. 5D). These results show that *Sp5* and *Sp8* are essential for NM cell development and that they may have additional redundant roles in anterior neural development.

In addition to being required for the maintenance of NM stem cells, *Wnt3a* plays a critical role in directing NM stem cells towards a paraxial mesoderm cell fate. In the absence of *Wnt3a* activity, presumptive paraxial mesoderm cells are thought to adopt neural fates and form ectopic neural tubes posterior to the last formed somite [10], [12]. To determine if *Sp5;Sp8* dko mutants also display ectopic neural tissue, we analyzed cross sections of mutant embryos for the expression of the neural marker Sox2. Indeed, a supernumerary *Sox2^+^* neural tube lying ventral to the primary neural tube was observed in *Sp5;Sp8* dko similar to *Wnt3a^−/−^*mutants (Fig. 5M–P, Q–T). Fibronectin staining was used to highlight embryonic structure and clearly shows that embryos that display ectopic neural tubes also lack somites (arrowheads, Fig. 5K, O, S). Thus the severe posterior abnormalities resulting from changes in neural and mesodermal fates are nearly identical in both *Sp5;Sp8* and Wnt3a mutants. These data show that like *Wnt3a*, *Sp5* and *Sp8* play a major role in determining NM stem cell fate.

## Discussion

We have characterized the *Wnt3a* transcriptome in the node and primitive streak (PS) at a developmental stage when the mammalian embryo is undergoing gastrulation, segmentation and axis extension. Our genomic data strongly supports a role for *Wnt3a* as a major regulator of PS stem cells, including NM stem cells. Many PS-specific genes identified in the microarray were down-regulated implicating *Wnt3a* as an important regulator of PS fate. The most significant sets of differentially expressed genes were associated with neural or paraxial mesoderm cell identity lending strong support to our hypothesis that *Wnt3a* directly regulates NM progenitors. Interestingly, neural-specific gene expression was marked by increased anterior and decreased posterior neural gene expression. In parallel, posterior paraxial PSM markers were also down-regulated while the anterior PSM markers, *Ripply2* and *Mesp2* increased. These findings suggest that Wnt3a suppresses anterior, and activates posterior, gene expression in both the neural plate and the PSM, consistent with a proposed role for Wnt/β-catenin signaling as the major evolutionarily conserved posteriorizing signal during the establishment and maintenance of axial AP polarity [35].

Wnt/β-catenin signaling plays a well-established role in the regulation of embryonic and adult stem cell homeostasis [36]. Somewhat paradoxically, Wnt/β-catenin signaling can promote both the maintenance of pluripotent ESCs as well as their differentiation into germ layer progenitors [32], [36]. Although our studies did not focus on pluripotency, *Klf5,* which encodes a transcription factor redundantly required for ESC pluripotency [37], was down-regulated in *Wnt3a* mutants suggesting that *Klf5* might regulate PS stem cell potency. This screen also led to the identification of the bHLH transcription factor, Msgn1, as a direct target gene of Wnt3a [19]. Msgn1 is a major activator of the oscillating segmentation clock - an activity that is a defining feature of PSM cells - suggesting that Msgn1 may be the key regulator of PSM differentiation.

*Sp5* and *Sp8* are expressed in numerous tissues throughout mouse development, [17], [38] but are restricted to important signaling centers where Wnts are active and where stem cells reside, such as the primitive streak. The orthologous genes *Sp5l* and *Sp5* have been implicated as important regulators of posterior development in zebrafish [39], [40], but a role for mammalian *Sp5* has not been clearly established. Although *Sp5* is not required for mammalian embryonic development, an *Sp5* null allele enhanced the short or kinked tail phenotypes in *T* [17] and *Wnt3a* heterozygotes suggesting it may indeed function in NM progenitors. The case for a role for *Sp8* in NM progenitors is stronger given that genetic ablation of *Sp8* resulted in the loss of all caudal (tail) somites and led to the development of neural anomalies [10], [14]. We have now shown, through the generation of *Sp5;Sp8* double mutants, that *Sp5* and *Sp8* have essential, overlapping roles in trunk and tail development. With the exception of the anterior exencephaly defects, the *Sp5;Sp8* dko phenotype is nearly identical to the *Wnt3a^−/−^* phenotype. Double mutants lack posterior paraxial mesoderm and display ectopic neural tubes, strongly suggesting that *Sp5* and *Sp8* function to regulate the fate of NM stem cells.

We have shown that Wnt3a/β-catenin signaling is both necessary and sufficient for *Sp5* and *Sp8* expression in the PS, PSM, and in ESCs. The high degree of similarity between *Wnt3a*, conditional β-catenin, *Tcf1;Lef1,* and *Sp5;Sp8* mutants suggests that Sp5/8 play equally important roles as β-catenin and Tcf1/Lef1 to transduce Wnt3a signals. This raises the intriguing question as to whether *Sp5/8* function as terminal effectors of a Wnt3a-initiated transcriptional cascade, or as novel, integral components of the β-catenin/Lef1 transcription complex. Alternatively, Sp5/Sp8 could function in a positive feedback loop to maintain *Wnt3a* expression. Future studies will address the precise molecular mechanisms of Sp5 and Sp8 activity in stem cells of the early embryo.

## Supporting Information

Figure S1 **Heat Map of all 729 DEGs between wt and** ***Wnt3a−/−*** **mutants (p<0.05).** Hierarchical clustering of all DEGs illustrates the consistency between replicates.
(TIF)

Figure S2 **Wnt/wg gene regulatory network.** Signal transduction pathway analysis (Genomatix, GePS software) identified 65 of the DEGs as members of the Wnt/wg gene regulatory network. Levels of differential gene expression are reflected by color and intensity: Green: down-regulated genes; Red: up-regulated genes Dashed lines highlight connections between network genes; diamonds indicate characterized and predicted promoter binding sites for connected factors; solid lines indicate interaction between proteins.
(TIF)

Figure S3 **Genetic interaction between** ***Sp5*** **and** ***Sp8*** **.**
(TIF)

Table S1 List of differentially expressed genes (DEG) determined by comparing transcriptional profiles of *Wnt3a−/−* and wildtype PS/node.
(XLSX)

Table S2 Signal transduction pathway analysis of DEG.
(XLS)

Table S3 Tissue-specific expression patterns associated with DEG.
(XLS)

Table S4 qPCR primers used to analyze *Sp1*-like family expression.
(DOCX)
