# Correction: Transcriptional Profiling of Wnt3a Mutants Identifies Sp Transcription Factors as Essential Effectors of the Wnt/β-catenin Pathway in Neuromesodermal Stem Cells

**DOI:** 10.1371/journal.pone.0095947

**Published:** 2014-04-18

**Authors:** The PLOS ONE Staff

The genotypes displayed in Figure 4 are incorrect. The genotype of the embryo depicted in Figure 4B should read Sp5lacZ/lacZ; Wnt3a+/+. The genotype of the tail shown at the top of Figure 4E is Sp5lacZ/+;Wnt3a+/+, and in Figure 4F, the second bar on the left represents the genotype Wnt3a+/-.

**Figure 4:**
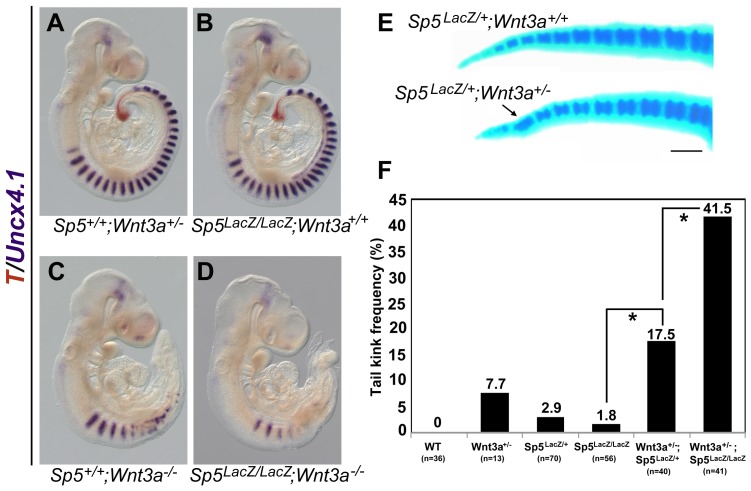
Genetic interactions between Sp5 and Wnt3a during tail development. (A-D) Two-color WISH illustrating Uncx4.1 (purple) and T (orange) in Sp5+/+;Wnt3a+/- (A), Sp5lacz/lacz;Wnt3a+/+ (B), Sp5+/+;Wnt3a-/- (C), and Sp5lacz/lacz;Wnt3a-/- double mutants (D) at E9.5. Double mutants (n = 8) display fewer and less-defined Uncx4.1 expressing anterior somites as compared to stage-matched Wnt3a null embryos. (E) At E18.5, Sp5lacz/+;Wnt3a+/- animals (bottom) display abnormal, fused tail vertebrae (arrow) compared to Sp5lacz/+;Wnt3a+/+ mice (top). (F) Frequency of tail kinks (expressed as %) observed in Wnt3a+/- heterozygotes at 6 weeks of age increased dramatically as the Sp5 gene dosage was reduced. The number of animals examined (n) is indicated below each genotype. *, statistical significance (p<0.0001, Chi-squared t-test). Scale bar  =  500um (E).

Please see the corrected Figure 4 here.
