# Supplementary figures and images for: Transcriptional Profiling of Wnt3a Mutants Identifies Sp Transcription Factors as Essential Effectors of the Wnt/β-catenin Pathway in Neuromesodermal Stem Cells
Source: PLoS One. 2014 Jan 24;9(1):e87018. doi: 10.1371/journal.pone.0087018 (PMC3901714; doi:10.1371/journal.pone.0087018)

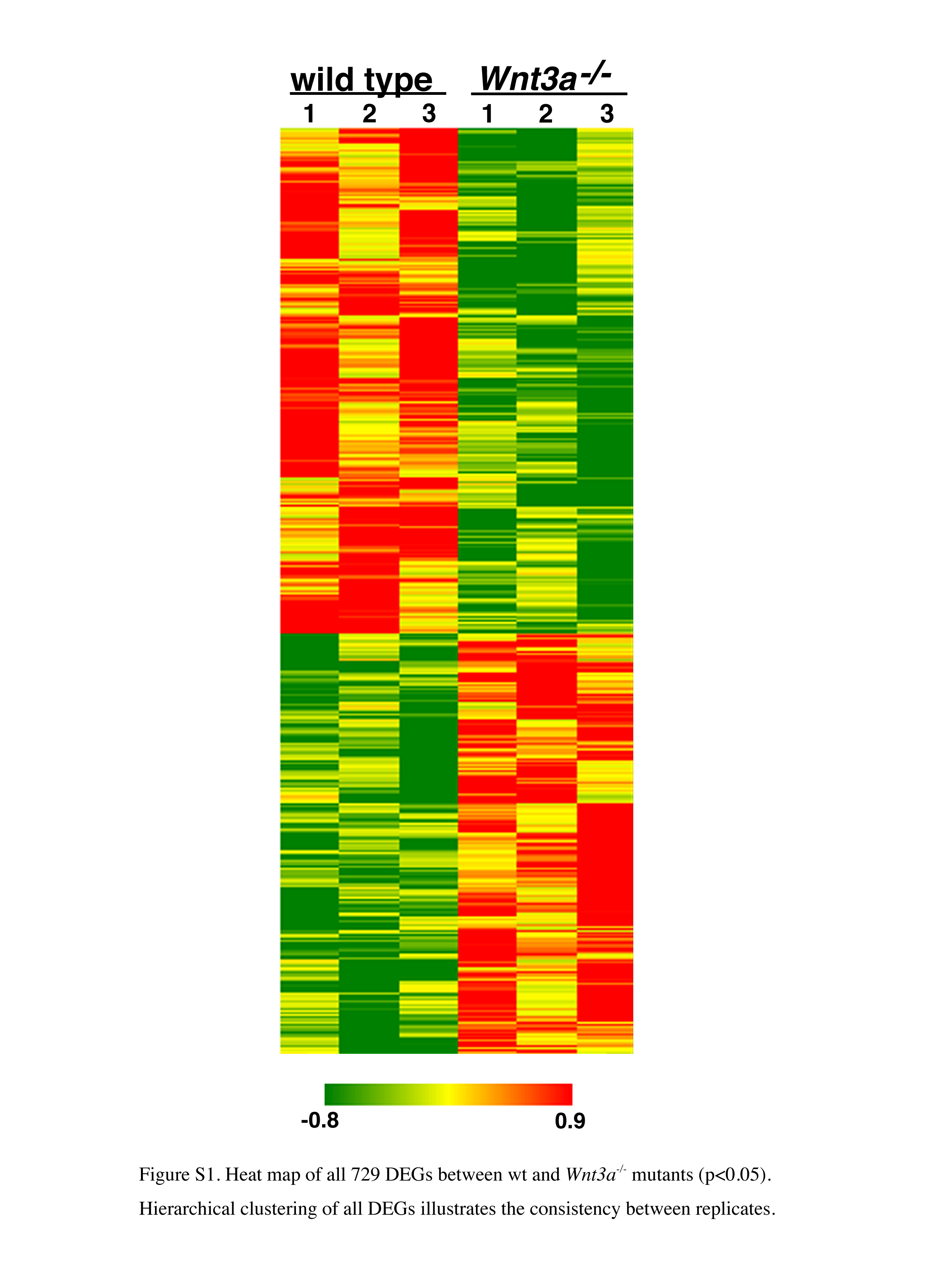

Supplement: Figure S1 — Heat Map of all 729 DEGs between wt and Wnt3a−/− mutants (p<0.05). Hierarchical clustering of all DEGs illustrates the consistency between replicates. (TIF) [file pone.0087018.s001.tif]

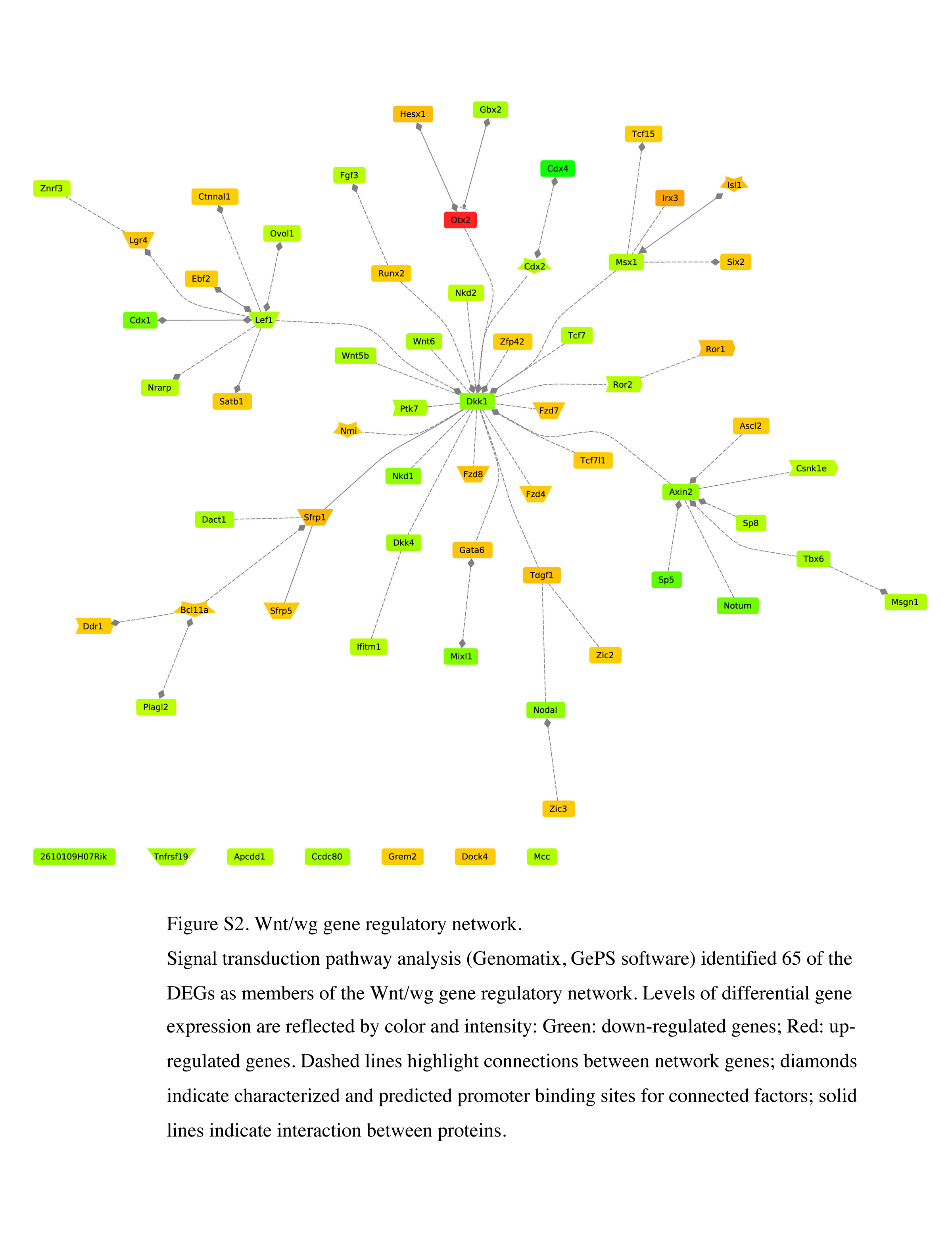

Supplement: Figure S2 — Wnt/wg gene regulatory network. Signal transduction pathway analysis (Genomatix, GePS software) identified 65 of the DEGs as members of the Wnt/wg gene regulatory network. Levels of differential gene expression are reflected by color and intensity: Green: down-regulated genes; Red: up-regulated genes Dashed lines highlight connections between network genes; diamonds indicate characterized and predicted promoter binding sites for connected factors; solid lines indicate interaction between proteins. (TIF) [file pone.0087018.s002.tif]

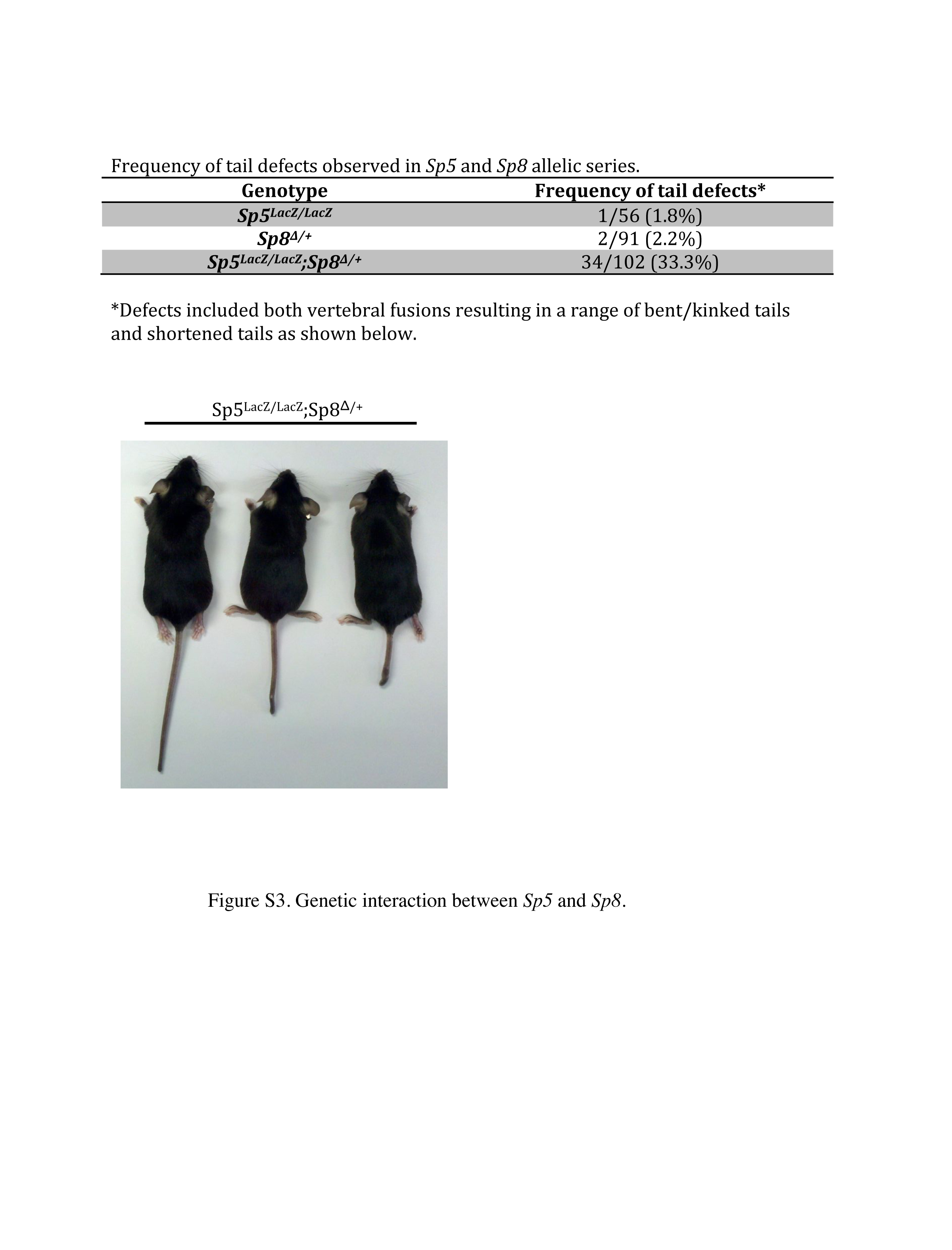

Supplement: Figure S3 — Genetic interaction between Sp5 and Sp8 . (TIF) [file pone.0087018.s003.tif]
