# Supplementary material for: Transcriptional Profiling of Wnt3a Mutants Identifies Sp Transcription Factors as Essential Effectors of the Wnt/β-catenin Pathway in Neuromesodermal Stem Cells
Source: PLoS One. 2014 Jan 24;9(1):e87018. doi: 10.1371/journal.pone.0087018 (PMC3901714; doi:10.1371/journal.pone.0087018)
Supplement: Table S4 — qPCR primers used to analyze Sp1-like family expression. (DOCX) [file pone.0087018.s007.docx]

Table S4. qPCR primers used to analyze Sp1-like family expression.

| Gene | Forward Primer (5’🡺3’) | Reverse Primer (5’🡺3’) |
| --- | --- | --- |
| Sp1 | AGGGTCCGAGTCAGTCAGG | TCGCTGCCATTGGTACTGTTG |
| Sp2 | CCAGCCTACCCCAAGGAAAC | GGGAGCCCTGAATCTGAAGTAT |
| Sp3 | TCAAGTAGTCGCTAATGTGCCT | GAACTTCCCGAGAGTCCCAAA |
| Sp4 | ATGAGCGATCAGAAGAAGGAGG | GAGTCCCTATTTTGCTGCAAGT |
| Sp5 | GTACGGCAAAACGTCGCAC | GAGGTGATCGCTTCGCATGA |
| Sp6 | CCTGCAACCTCTCCAGACATA | GCCCTGTGAAAAGTCTACCTCC |
| Sp7 | ACCCCAAGATGTCTATAAGCCC | AGTTGCCCACTATTGCCAACC |
| Sp8 | GCCAACGACTACTCTGTGTTC | GGGAGCTATCCTGCGAGTG |
| Sp9 | GCTTCCACTCGACCCTAGC | GAGCTGAAGTCGGGGTTGTA |
| Gapdh | AGGTCGGTGTGAACGGATTTG | TGTAGACCATGTAGTTGAGGTCA |
